# A pilot study of a sexual health workshop for oncology staff: feasibility, impact, and gaps in addressing young adult breast cancer survivorship needs

**DOI:** 10.1007/s11764-026-01985-8

**Published:** 2026-03-16

**Authors:** T. J. Rick, A. Girard, A. Topalian, J. Hui, R. J. Jansen, A. H. Blaes

**Affiliations:** 1 Department of Medicine, Division of Hematology, Oncology, and Transplantation, University of Minnesota, Minneapolis, MN USA; 2 University of Minnesota Masonic Cancer Center, Minneapolis, MN USA; 3 Eli Coleman Institute for Sexual and Gender Health, Department of Family Medicine and Community Health, University of Minnesota, Minneapolis, MN USA; 4 Department of Family and Community Medicine, Division of Survivorship and Supportive Services, University of Cincinnati, Cincinnati, OH USA; 5 Department of Surgery, Division of Surgery, University of Minnesota, Minneapolis, MN USA; 6 Biostatistics Core, Masonic Cancer Center, University of Minnesota, Minneapolis, MN USA

**Keywords:** Sexual health, Adolescent and young adult (AYA), Cancer survivors, Oncosexology, Sexual health, Psychosexual, Breast cancer, Quality of life, Screening, Training workshop

## Abstract

**Purpose:**

Sexual health concerns are highly prevalent and distressing among adolescent and young adult (AYA) breast cancer survivors, yet are rarely addressed in routine oncology care. Lack of provider training remains a key barrier to effective communication and support. The purpose of the study was to determine whether a brief educational workshop for oncology staff improves sexual health screening rates among AYA breast cancer survivors, and evaluate the workshop’s acceptability, feasibility, and impact on provider confidence.

**Methods:**

We conducted a pilot multi-component study combining a mixed observational cohort and non-randomized interventional study. On May 20, 2024, eight breast cancer care team members—four advanced practice providers (APPs) and four registered nurse care coordinators (RNCCs)—participated in a “30-s message” communication workshop adapted from the *All of Me* program created by After Cancer. Medical charts of all eligible AYA breast cancer survivors aged 18–40 diagnosed within the previous 24 months were reviewed to assess changes in sexual health screening documentation before and six months post-intervention. Provider confidence, perceived importance of sexual health, and perceptions of feasibility and acceptability were evaluated via pre-, post-, and 6-month follow-up surveys.

**Results:**

Twenty-two AYA breast cancer survivors were eligible for chart extraction. Prior to the intervention, 45% of patients had documented sexual health screening, with APPs responsible for nearly all documented cases. Screening rates did not increase following the workshop. Of the 12 patients not screened, 4 transferred care (33%), 5 saw an oncologist (41%), 1 saw an APP (8%), and 2 had other competing health issues occurring at the time of follow up that took priority of the visit (17%). All eight participating providers (4 APPs, 4 RNs) completed the training; 88% completed the immediate post-survey and 75% completed the six-month follow-up survey. Providers reported high acceptability and feasibility. Confidence in addressing physical symptoms was higher than managing emotional concerns (e.g., low desire).

**Conclusions:**

Screening rates did not increase following the workshop, likely reflecting high baseline APP screening and limited follow-up opportunities. Future efforts should integrate role-specific education with system-level supports to broaden and sustain screening practices.

**Implications for Cancer Survivors:**

Cancer survivors are often cared for by a multidisciplinary team. This study suggests that training only advanced practice providers and nurses may not be enough to improve sexual health screening. Broader education across all care team members, combined with system-wide tools and prompts, may be needed to ensure consistent and equitable survivorship care.

## Introduction

In the USA, there are over 18 million cancer survivors, and this number continues to rise. Adolescent and young adult (AYA) cancer survivors, aged 15–39 years, are an important population with unique needs [1]. Approximately 90,000 AYA individuals are diagnosed with cancer annually, representing 4.2% of new cancer diagnoses in 2025, of which approximately 11,000 (12%) have a breast cancer diagnosis [2–4]. Nearly half of the AYA population, and up to 90% of breast cancer survivors report detrimental effects on their sexual health, resulting in a negative impact on quality of life [5–8]. Sexual health includes physical, psychosocial, and developmental factors, all of which may have adverse impacts from cancer and it’s treatment (B. [9]). Sexual dysfunction in AYA survivors is often long lasting [10]. Prior studies document AYA patients report higher rates of sexual dysfunction, often accompanied by lower satisfaction in sex life and delays in meeting sexual milestones when compared to older cancer survivors and healthy peers [11]. However, limited information is available to inform the care and management of sexual health issues in AYA cancer survivors [9].

Cancer survivorship care is designed to mitigate the effects of cancer and cancer treatment to improve quality of life. However, sexual health is an increasingly recognized unmet need in cancer survivorship care and there is a communication gap between health care providers and patients. Although the American Society of Clinical Oncology (ASCO) recommends that a member of the oncology team screen patients for sexual dysfunction and the National Comprehensive Cancer Network (NCCN) provides management guidelines, this is not regularly occurring in practice, particularly among female survivors [12–16]. Additionally, while guidelines recommend discussing and screening for sexual dysfunction, specific psychosexual interventions and strategies to incorporate screening into clinical care are lacking [17]. Patients are not initiating the conversation about sexual health yet want someone from the health care team to address their concerns [18–20]. A prior study of AYA survivors found participants would like their health care team to initiate conversations and communicate openly and honestly about sexual health without taboo, bias, or shame [21]. Lack of provider experience and appropriate training on how to screen and address sexual health concerns is one of the greatest barriers to care. However, a majority of medical providers report interest in acquiring additional training to address this knowledge gap [22]. Young breast cancer survivors specifically represent a distinct group facing a complex burden of sexual health issues due to prolonged treatment exposure and developmental life stage. Despite elevated risk, these concerns often go unaddressed, and addressing these gaps is critical for long-term adherence and survivorship quality.

Advanced practice providers (APPs), a term encompassing physician associates (PAs) and nurse practitioners (NPs), along with registered nurse care coordinators (RNCCs) and other oncology team members, play a critical role in delivering high-quality cancer care. These roles are often centered around patient education, side effect management, and quality of life of the survivor [23]. We utilized the “30-s message” workshop that trains medical professionals to create their own introductory message about sexual health with the purpose of normalizing the topic between patients and health care providers and inviting a conversation about sexual health.

The aims of this study were to (1) determine if the sexual health workshop for breast cancer medical professionals increases sexual health screening in young adult breast cancer survivors by conducting a retrospective cohort analysis of AYA breast cancer survivors seen in the 6-month period before and after the intervention and (2) determine if the workshop is perceived as important, acceptable, feasible, and increases the confidence of breast cancer medical professionals in screening patients for sexual health concerns.

## Methods

### Study design and population

We conducted a pilot mixed observational and non-randomized interventional study. The study includes two arms with different participant populations. The first arm was observational and included all eligible *breast cancer survivors*, stage I–IV, age 18–40 diagnosed between January 1, 2022–December 31, 2022, and were seeing one of the six breast oncologists at the Masonic Cancer Clinic, an urban oncology clinic at an NCI designated cancer center. The second arm was interventional with eight eligible APPs and RNCCs, i.e., *medical professionals*, employed within the breast cancer disease team in 2024. Breast cancer survivors were excluded if they transferred care, were lost to follow up (missed two or more routine follow up visits), opted out of research, or had a documented visit with a sexual health specialist where they already received sexual health screening. Medical professional participants were required to be over 18 years of age, English speaking, willing to participate in the workshop, and be mentally/physically competent to complete a questionnaire and provide implied consent. The study was approved by the University of Minnesota Institutional Review Board with exempted status (IRB # STUDY00021998) and implied consent was obtained for all medical professionals.

### Recruitment

The breast cancer survivor cohort was extracted from the Breast Cancer Registry by the Best Practices Integrated Informatics Core (BPIC), and the study team screened for eligibility criteria. There were eight breast cancer site team medical professionals, four APPs, and four RNCCs (the entire group of these professionals) identified and invited to participate in the workshop via email from the principal investigator. The workshop took place during normal work hours, and participants were excused from their clinical duties during this time.

### Intervention

We selected the All of Me Iowa program created by nonprofit After Cancer as the foundation for our pilot training based on its evidence-informed approach and alignment with the goals of our project. We are not paid affiliates and have no financial relationship with the organization. After Cancer is an educational initiative that aims to bridge communication gaps between health care providers and patients around sexual health. Its goals include normalizing sexual health discussions, setting clear patient expectations about treatment-related impacts, and outlining referral pathways for care. The program was developed in collaboration with oncology specialists and researchers at the University of Iowa and has been used to train diverse cancer care professionals, including oncology nurses, physical therapists, social workers, and APPs. In an unpublished pilot study with 24 rural oncology professionals, the training improved providers’ perception of the importance of sexual health and their confidence in addressing it. We adapted their “30-s message” tool for our context to promote feasible and standardized integration of sexual health screening into routine oncology care.

The workshop aims to help medical professionals create their own introductory message about sexual health with the purpose of normalizing the topic between the patients and health care providers and inviting a conversation about sexual health. The After Cancer facilitator (a cancer survivor) assisted participants in writing and practicing their own 30-s message with six key messages: (1) sexual health side effects are common and expected, (2) negative changes in sexual health affect quality of life, (3) sexual health is a medical concern, (4) there are resources to help, (5) this may not be a priority at the moment, and (6) addressing sexual health concerns sooner may make a difference on outcomes. An example of such a “30-s message” includes:

*I want to take a moment to ask you about sexual health, because changes in sexual function are very common and expected during and after cancer treatment. These changes can affect quality of life, and sexual health is an important medical concern, just like fatigue or pain.*

If the patient indicates no concerns or expresses that sexual health is not a current priority, a suggested follow-up phrase might include:

*I know this may not feel like a priority right now, and that’s okay, but I want you to know there are resources and treatments available if this becomes an issue. Addressing concerns earlier can sometimes make a difference, so if you ever notice changes or have questions, please let me know and we can talk about options or connect you with support.*

Participants were given patient education handouts in English and Spanish, and other resources from the After Cancer website [24].

At the completion of the “30-s message” workshop (approximately 90 min), the principal investigator reviewed the NCCN Survivorship guidelines for Sexual Health, local resources and referrals available to support patients, and templated phrases that could be used in the electronic health record (EHR) to capture sexual health screening. Additional education was provided on the management of four common diagnoses: (1) genitourinary syndrome of menopause, (2) hypoactive sexual desire disorder, (3) dyspareunia, and (4) anorgasmia in order to help participants facilitate discussion, EHR documentation, and provide patient education.

### Study procedures

#### Primary outcome

Prior to the workshop intervention, a chart abstraction of eligible breast cancer survivors was performed, including demographics, pertinent cancer treatment information, and presence or absence of sexual health screening conducted by a member of the breast cancer disease team. Using the search function in the EHR, keywords—sexual health/dysfunction, urogenital/genitourinary symptoms, vaginal dryness, dyspareunia, painful intercourse/sex/penetration, low libido/desire, orgasm, and anorgasmia—were evaluated to find any sexual health screening documentation. The health care team member (RN, APP, MD, etc.) who performed the screening was recorded. Six months after the workshop, the same cohort of patients was re-evaluated for sexual health documentation in the same fashion. The 6-month follow-up period was selected based on standard surveillance guidelines recommending patient visits every 3 months, with care typically alternating between MDs and APPs, thus allowing for at least one APP visit in which screening behavior could be evaluated post-intervention.

#### Secondary outcomes

**Fig. 1:**
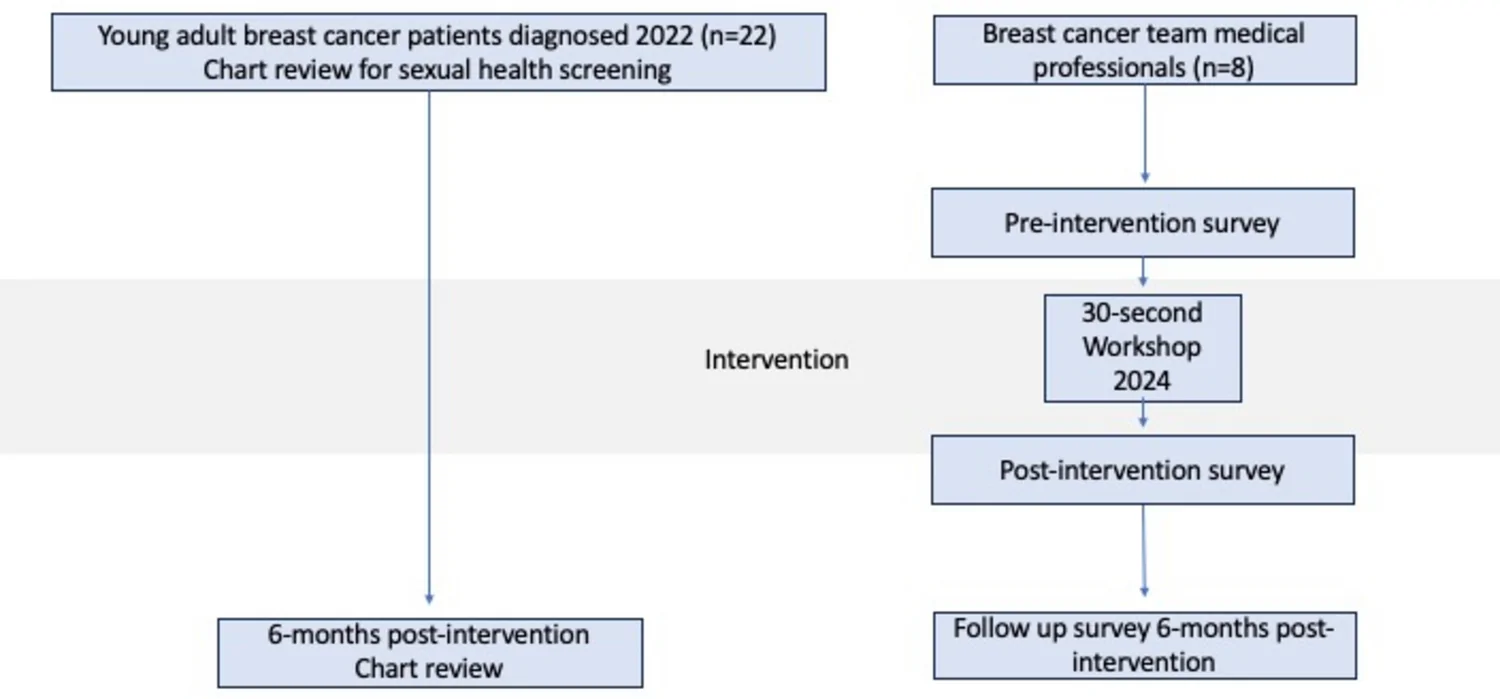
Study schema

Medical professionals were surveyed about the workshop. Key measures included (1) perception of importance of sexual health, (2) acceptability, (3) feasibility of applying the concepts into clinical practice, and (4) confidence discussing different components of sexual health with patients. We assessed the perception of the importance of screening patients for sexual health concerns before and after the training. Acceptability and feasibility of the workshop were evaluated with a validated Intervention Appropriateness Measure (IAM) immediately after the intervention [25, 26]. Three open-ended questions were included for participants to elaborate on aspects of acceptability (i.e., asking participants what they liked, disliked, or would change about the workshop). Confidence was measured by participant’s perceived comfort in engaging in three key communication tasks: (1) initiating a conversation about sexual health, (2) offering information about the impact of breast cancer treatment(s) on sexual health, and (3) providing a referral for sexual problems when appropriate [14, 15, 27]. Questions were rated on a 5-point Likert scale. Measures were collected via REDCap online surveys at baseline, post-intervention (same day), and 6-month post intervention. Study schema is provided in Fig. 1.

### Statistical considerations

Twenty breast cancer survivors were evaluated to detect a difference in the proportion of those screened for sexual health concerns before and after intervention. To compare the proportions of sexual health screening of breast cancer survivors at pre- and post-intervention, McNemar’s test was used. R statistical program was utilized to assess the changes and visualize study data. Data summaries were created showing the mean and standard deviation for continuous variables and the number and percentage for categorical variables. Survey data were reported on the Likert scale and summarized using percentages in each category. Statistical tests were reported with corresponding p-values, with a significance cutoff of 0.05.

## Results

### Primary outcome- sexual health screening

A total of 27 breast cancer survivors were eligible for chart abstraction, and 22 met eligibility criteria (five patients either being lost to follow up or transferred care). Pre-intervention, 10/22 (45%) were screened for sexual health concerns. Post-intervention, no additional patients were screened for sexual health concerns. Estrogen receptor (ER) positive patients (Table 1) and patients on gonadotropin-releasing hormone (GNRH) agonists and aromatase inhibitors (AIs) were more likely to get screened (Table 2). At baseline, patients were more likely to be screened by an APP than by a physician (6 of 10, 60% vs. 4 of 10, 40%), though this difference was not statistically significant (*p* = 0.4). Of the 12 patients not screened, 4 transferred care (33%), 5 saw an oncologist (41%), 1 saw an APP (8%), and 2 had other competing health issues occurring at the time of follow up that took priority of the visit (17%).

**Table 1:** Comparison of demographic and clinical characteristics of breast cancer patients: screened vs. non-screened for sexual health concerns *N* = 22

Characteristic	Not screened *n* = 12^*1*^	Screened *n* = 10^*1*^	*p*-value^*2*^
Age	36.5(4.8)	36.6(3.8)	> 0.9
Stage			0.8
1	3 (25%)	5 (50%)	
2	5 (42%)	3 (30%)	
3	1 (8.3%)	1 (10%)	
4	2 (17%)	1 (10%)	
Local recurrence	1 (8.3%)	0 (0%)	
Treatment intent			> 0.9
Curative	10 (83%)	9 (90%)	
Palliative	2 (17%)	1 (10%)	
Molecular subtype			0.014
ER-/HER2-	4 (33%)	0 (0%)	
ER-/HER2+	2 (17%)	0 (0%)	
ER+/HER2-	3 (25%)	9 (90%)	
ER+/HER2+	3 (25%)	1 (10%)	
Type of surgery			0.7
Bi-mastectomy	6 (50%)	4 (40%)	
Declined	1 (8.3%)	0 (0%)	
Lumpectomy	3 (25%)	3 (30%)	
None	1 (8.3%)	0 (0%)	
Uni-mastectomy	1 (8.3%)	3 (30%)	

^*1*^Mean (SD); *n* (%)

^*2*^Wilcoxon rank sum test; Fisher’s exact test

**Table 2:** Comparison of treatment characteristics of breast cancer patients: screened vs. non-screened for sexual health concerns *N* = 22

Characteristic	Not screened *n* = 12^*1*^	Screened *n* = 10^*1*^	*p*-value^*2*^
Radiation			0.2
N	9 (75%)	4 (40%)	
Y	3 (25%)	6 (60%)	
Chemotherapy			> 0.9
N	1 (8.3%)	1 (10%)	
Y	11 (92%)	9 (90%)	
Oophorectomy			> 0.9
N	10 (83%)	8 (80%)	
Y	2 (17%)	2 (20%)	
GNRH agonist			0.006
N	11 (92%)	3 (30%)	
Y	1 (8.3%)	7 (70%)	
AI			0.004
N	9 (75%)	1 (10%)	
Y	3 (25%)	9 (90%)	
Tam/SERM			0.5
N	12 (100%)	9 (90%)	
Y	0 (0%)	1 (10%)	
Targeted therapy			0.2
N	3 (25%)	6 (60%)	
Y	9 (75%)	4 (40%)	

^*1*^*n* (%)

^*2*^Fisher’s exact test

### Secondary outcomes—importance, acceptability, feasibility, and confidence

Eight breast cancer medical professionals were invited and participated in the intervention. All completed the pre-intervention survey, 7/8 (88%) completed the post-intervention survey, and 6/8 (75%) completed the 6-month follow-up survey. All eight participants identified as women, with a majority over the age of 50 years, with less than four years of oncology experience, and without prior sexual health training (Table 3).

**Table 3:** Demographic and professional characteristics of oncology health care professionals participating in a sexual health communication workshop (*N* = 8)

Characteristic	*N* = 8^*1*^
Profession	
Physician assistant	3 (38%)
Nurse practitioner	1 (13%)
Registered nurse	4 (50%)
Gender identity	
Woman	8 (100%)
Age	
20–29	1 (13%)
30–39	1 (13%)
40–49	1 (13%)
50–59	5 (63%)
Years of experience in oncology	
1–4	4 (50%)
5–9	1 (13%)
10–14	1 (13%)
15–19	2 (25%)
Sexual health training in hours	3 (38%)
1–4	2 (67%)
5–9	1 (33%)
Unknown	5

^*1*^*n* (%)

#### Importance

**Fig. 2:**
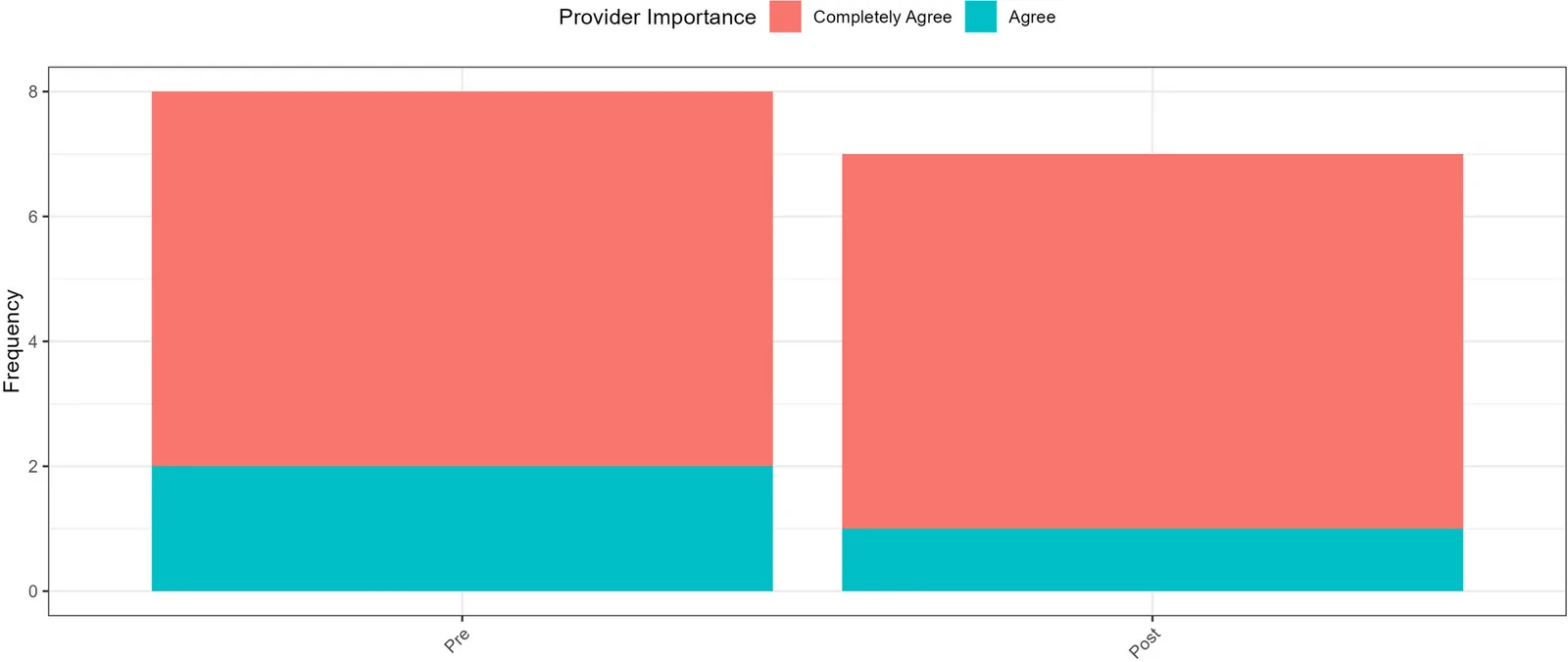
Stacked bar graphs showing provider importance of screening in relationship to intervention. The y-axis presents frequency, x-axis shows the pre and post intervention timepoints, and color represents category of response. This plot is able to illustrate temporal shifts in screening importance across time

Participants felt the topic was important at baseline and post intervention (Fig. 2).

#### Acceptability

**Fig. 3:**
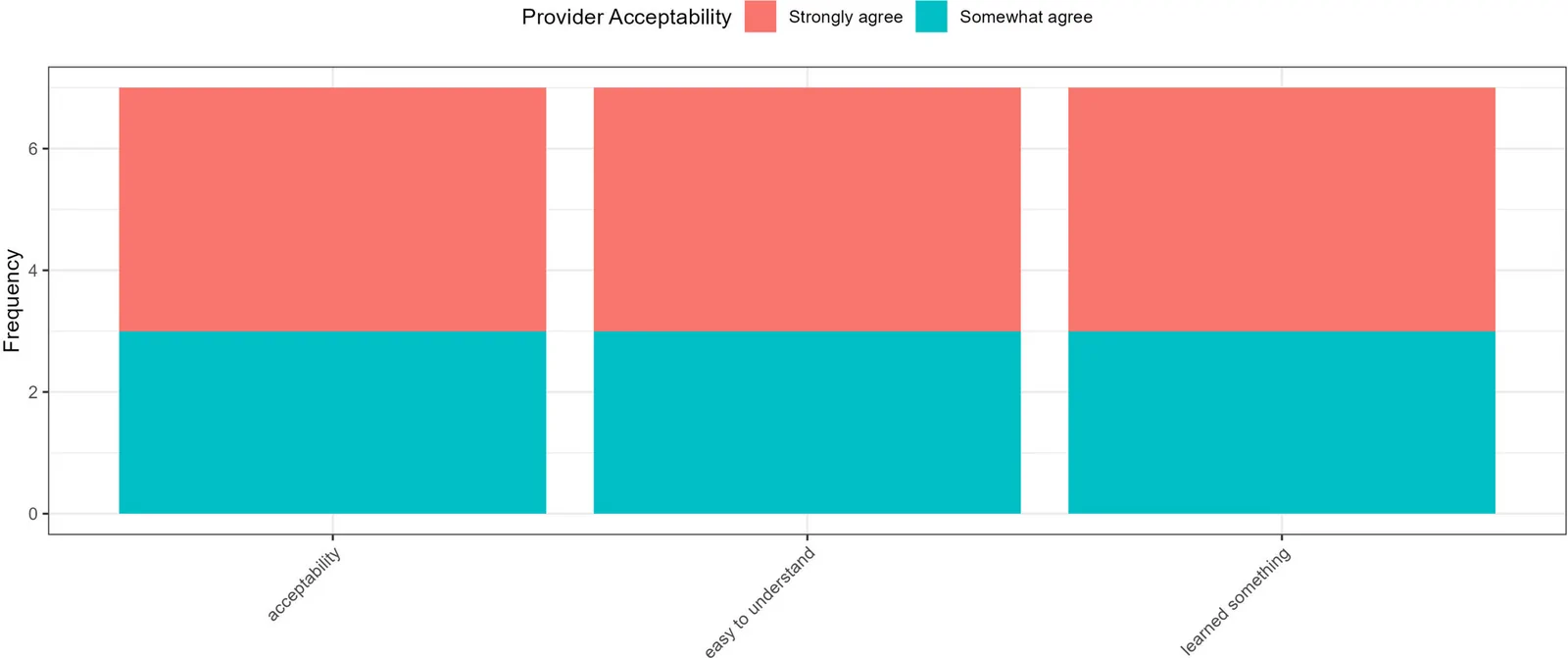
Stacked bar graphs showing provider acceptability responses regarding intervention. The y-axis presents frequency, the x-axis is split based upon different acceptability questions, and color represents category of response. This plot illustrates provider acceptability to the educational intervention

There was a high level of positive feedback on the workshop, suggesting it was well received, easily understood, and informative (Fig. 3). Participants reported that the workshop was “presented in a very understandable way,” “interactive, to the point.” Other feedback included “I liked the small group,” and “I left with a plan to improve patient care.” Three participants reported they liked receiving a patient perspective. Two participants left wanting more information.

#### Feasibility

**Fig. 4:**
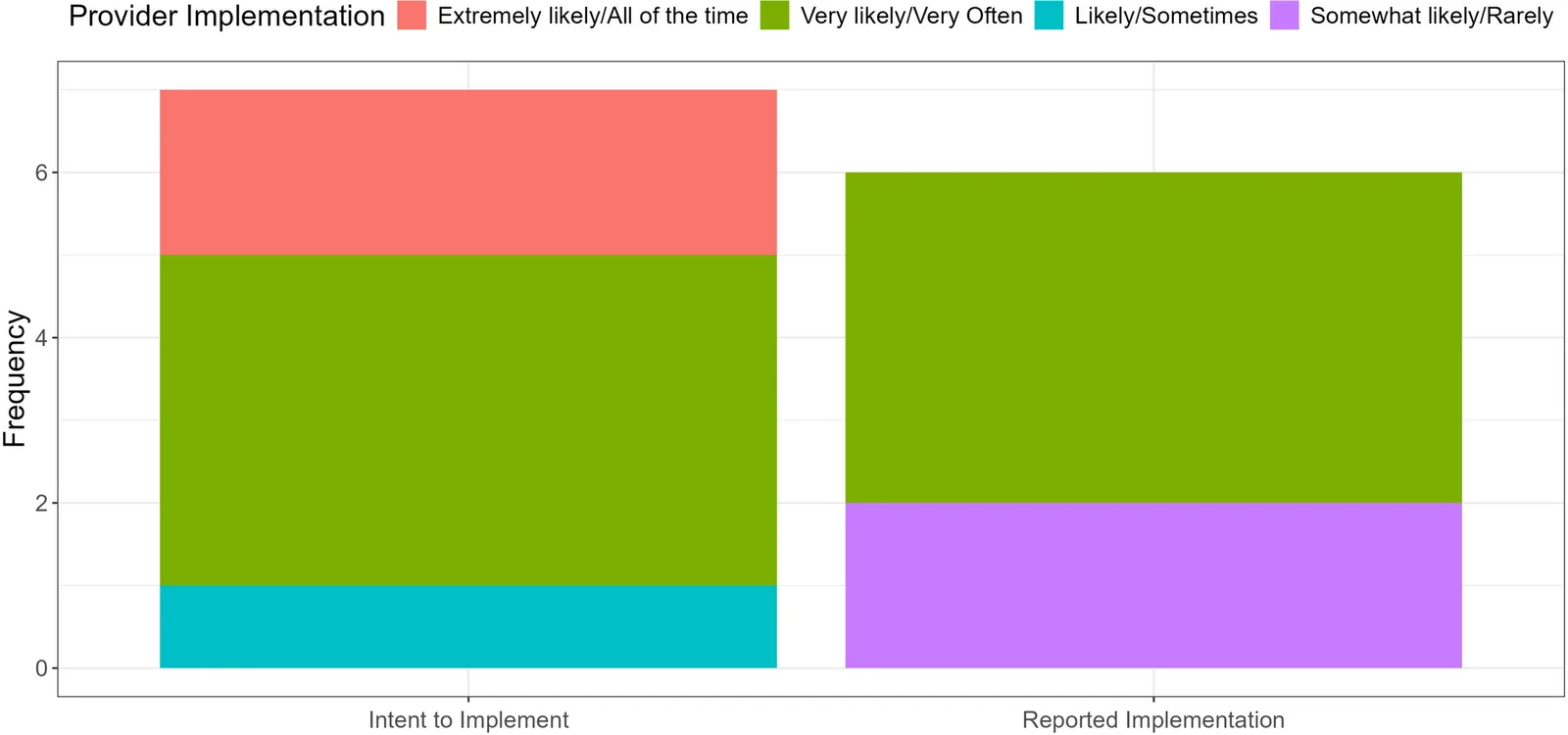
Stacked bar graphs showing intent and actual providers’ implementation of screening in practice. The y-axis presents frequency, and the x-axis categorizes the responses to questions related to intent and actual implementation, and color represents the category of implementation. This plot is able to illustrate a comparison between intent and practice

The feasibility of implementing the 30-Second Message into practice was rated highly (71%). However, at the 6-month follow-up, 33% of participants (all RNCCs) reported rarely implementing sexual health screening into their practice (Fig. 4). A follow-up question regarding barriers to consistent implementation in clinical practice was intended; however, due to a survey administration error, it was not included.

#### Confidence

**Fig. 5:**
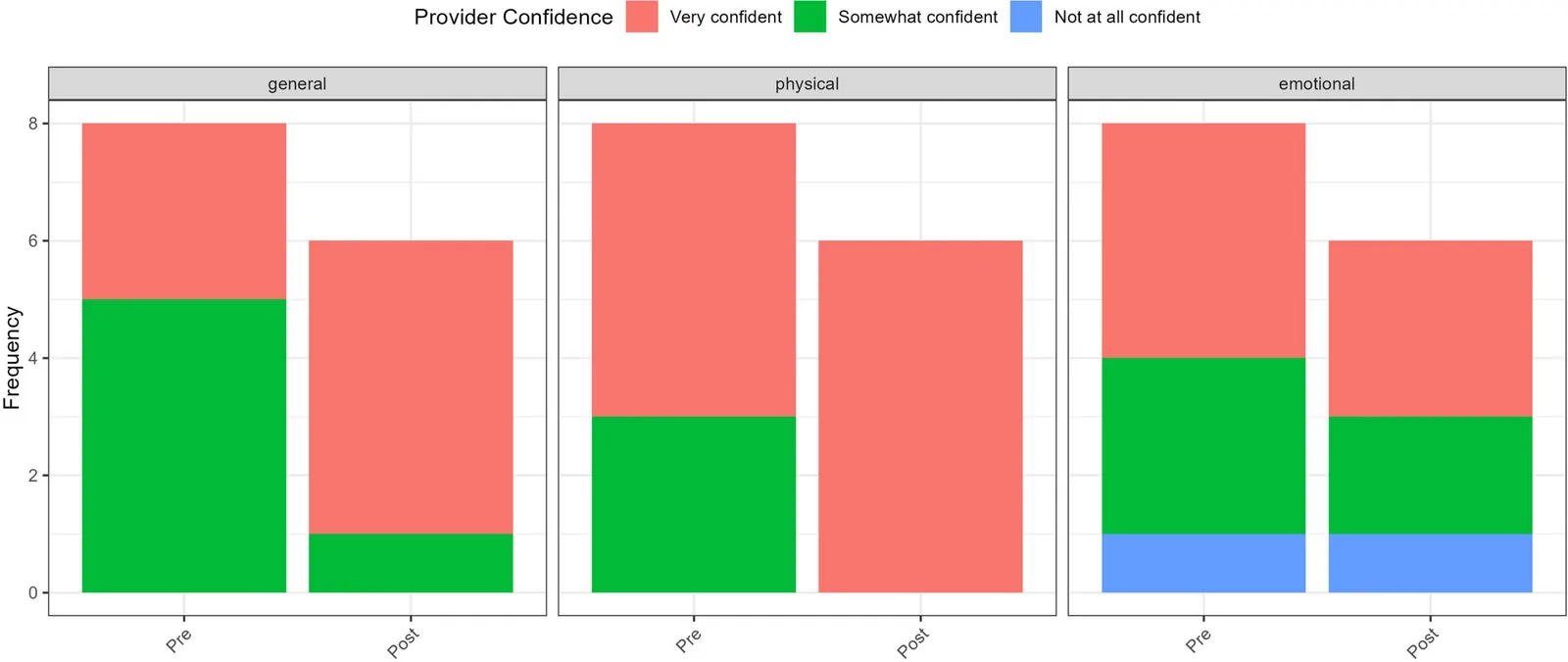
Stacked bar graphs showing providers’ confidence in different domains collected pre and post educational intervention. The y-axis presents frequency, the x-axis groups responses into pre and post intervention timepoints, and color represents confidence category. This plot is able to illustrate temporal shifts in confidence across time within each domain

Confidence increased regarding knowledge of general and physical concerns, suggesting a positive impact of the workshop. However, there was no change in confidence related to addressing emotional concerns (e.g., desire/libido) following the workshop. The apparent percentage difference reflects the lower response rate at six months, with 6 of 8 participants completing the follow-up survey. For example, 1 of 8 (12%) participants at baseline and 1 of 6 (17%) at follow-up reported being “not at all confident” in addressing the emotional aspects of sexual health (Fig. 5).

## Discussion

The primary aim of this pilot study was to evaluate the impact of an educational workshop on sexual-health screening practices among oncology staff caring for adolescent and young adult (AYA) breast cancer survivors, with a secondary aim of assessing the workshop’s overall efficacy. The workshop was well received but did not lead to increased screening rates in this small study. The APP participants in this pilot reported baseline confidence in addressing sexual health and were providing sexual health screening pre-intervention, suggesting they may not have been the ideal population to assess the impact of this particular educational workshop. At baseline, nearly 50% of patients had documented sexual-health screening, higher than rates reported in prior studies (30–40%) [15, 28]. Importantly, the absence of new screenings in the follow-up period does not imply a lack of willingness among trained providers. Rather, the majority of unscreened patients had not seen an APP or had follow-up visits focused on other pressing concerns. This highlights the importance of integrating screening prompts into diverse clinical contexts and across provider types, including medical oncologists who were not included in the current training. RNCCs also did not screen any patients during the follow-up period. This may reflect both structural and role-based limitations, including lower self-reported confidence and the nature of RNCC interactions, which often occur during periods of high treatment intensity (e.g., new diagnosis, chemotherapy initiation, surgical recovery) or through telephone encounters, settings less conducive to initiating sensitive or relational conversations. Moreover, barriers to screening in this group may not stem from lack of willingness to engage, but rather from uncertainty about how to manage a positive screen. Prior research in gynecologic oncology similarly reports that providers often hesitate to initiate discussions around sexual health when they feel ill-equipped to offer clear treatment pathways or referrals (Girard et al., in progress). Notably, patients have expressed interest in receiving sexual-health information regardless of provider type, as long as it is brought up in a respectful and timely manner [29, 30]. This highlights the need for role-specific training that not only emphasizes communication skills, but also equips providers with practical strategies for navigating and managing common sexual health concerns.

Certain patient characteristics were associated with lower screening rates, including estrogen receptor (ER)–negative disease and metastatic disease. This highlights missed opportunities with specific breast cancer subpopulations that are at risk for sexual dysfunction, body image concerns, and interpersonal distress. This is important as the literature reports few patients received any information up front about potential sexual effects of breast cancer treatment [31–33].

This study highlights lower confidence in addressing the emotional dimensions of sexual health, particularly low desire, a concern especially relevant for adolescent and young adult (AYA) cancer survivors. These individuals frequently experience complex and enduring sexual dysfunction driven by both physical and psychosocial factors, with reported prevalence ranging from 30 to 100% during and after treatment [9, 17]. Unlike older adults, AYAs are navigating critical developmental milestones, such as body image, sexual identity, and evolving intimate relationships, rendering their sexual health needs uniquely sensitive and often overlooked [17]. Failure to address these needs can compound distress, lead to social isolation, and negatively impact long-term quality of life [30]. A biopsychosocial and contextual framework is therefore essential to guide care [34]. Effective support requires providers who can normalize sexual health discussions, offer guidance and resources, and initiate referrals when appropriate (Lehmann et al., 2025b). However, fewer than half of oncology fellowship program directors offer formal training in sexual health communication, and most APPs report acquiring these skills on the job (Reese et al., 2024). Evidence-based interventions that strengthen patient-provider communication have been shown to improve both sexual function and quality of life [30], and should be coupled with tailored, actionable approaches that address both physical and emotional aspects of dysfunction (Lehmann et al., 2025b).

Consistent screening for sexual health concerns remains a critical, yet under-implemented component of comprehensive oncology care. Despite recommendations from national guidelines, routine sexual health screening is not widely adopted in clinical practice. Given the considerable time constraints inherent in oncology care, shifting from reliance on individual provider-initiated conversations to routine, standardized sexual health screening may help normalize assessment and reduce the burden on both patients and clinical staff. The integration of standardized tools, such as incorporating sexual health prompts into existing instruments like the NCCN Distress Thermometer, offers a systematic and scalable approach to identifying sexual dysfunction across diverse care settings. Adolescent and young adult (AYA) cancer survivors are particularly vulnerable to unmet sexual health needs due to their developmental stage and extended survivorship trajectory. Normalizing these often-overlooked conversations through standardized assessment could promote equitable identification of concerns and ensure these needs are not left unaddressed [17]. Moreover, such tools may help address persistent barriers cited by oncology providers, including limited time, inadequate training, and discomfort initiating sensitive conversations. To further support implementation, informatics-based solutions, such as clinical decision support tools, could assist providers in translating screening results into actionable, evidence-based management strategies for common concerns, including genitourinary syndrome of menopause and low sexual desire. These digital tools have been associated with improved clinical outcomes in other domains and represent a promising, scalable strategy for enhancing sexual health care in oncology settings [35–38].

This study has several limitations that warrant consideration. First, the sample size was small, comprising a limited number of patients and oncology staff, which may restrict the generalizability of findings. The study was conducted within a single urban, academic health system, and the results may not reflect screening practices or implementation barriers in other institutional or community oncology settings. Additionally, the intervention targeted only advanced practice providers (APPs) and registered nurse care coordinators (RNCCs); physicians were not included in the workshop, which may have influenced observed screening rates, especially if sexual health was traditionally addressed or omitted in physician-led visits. We selected a 6-month follow-up interval for chart abstraction based on expected breast cancer surveillance care patterns, in which patients are seen every 3 months and alternate between APP and physician visits. However, variability in follow-up schedules and care transitions may have limited opportunities for trained providers to apply workshop skills. Finally, as a non-randomized, single-arm pilot, the study was not designed to determine causality, and findings should be interpreted as exploratory and hypothesis generating.

Although the brief educational workshop was well received by participating APPs and nurses, it did not result in increased documentation of sexual health screening. High baseline screening rates among APPs and the limited number of eligible follow-up visits may have contributed to the null findings. However, most patients not screened during follow-up were seen by providers who had not participated in the training, suggesting that targeting only one segment of the care team may be insufficient. Future efforts to improve sexual health communication in cancer care should consider role-specific education across the entire oncology team—including physicians—alongside the implementation of system-level tools such as embedded prompts, standardized workflows, and evidence-based clinical support resources. These strategies may help normalize sexual health discussions and support consistent, equitable survivorship care in busy clinical settings.

## Data Availability

No datasets were generated or analysed during the current study.

## Declarations

### Disclaimer

All statistical analyses were carried out in the Biostatistics Shared Resource of the Masonic Cancer Center, supported in part by National Cancer Institute Cancer Center Support grant P30CA077598 and the National Center for Advancing Translational Sciences of the National Institutes of Health Award Number UM1TR004405. The content is solely the responsibility of the authors and does not necessarily represent the official views of the National Institutes of Health.
